# Severe maternal morbidity by mode of delivery in women with twin pregnancy and planned vaginal delivery

**DOI:** 10.1038/s41598-020-61720-w

**Published:** 2020-03-18

**Authors:** Diane Korb, Catherine Deneux-Tharaux, François Goffinet, Thomas Schmitz

**Affiliations:** 1 Université de Paris, Epidemiology and Statistics research Center/CRESS, INSERM, INRA, F-75004 Paris, France; 2 Department of Obstetrics and Gynecology, Robert Debré Hospital, APHP, Paris, France; 3 Port-Royal maternity unit, Cochin Hospital, APHP, Paris, France

**Keywords:** Risk factors, Fever

## Abstract

Planned vaginal delivery in twin pregnancies has three potential outcomes: vaginal or cesarean delivery of both twins, or cesarean for the second twin. Our objective was to assess the association between delivery mode and severe acute maternal morbidity (SAMM) in women with twin pregnancies and planned vaginal deliveries. We limited this planned secondary analysis of the JUMODA cohort, a national prospective population-based study of twin deliveries, to women with planned vaginal delivery at or after 24 weeks of gestation who gave birth to two live fetuses at hospital. The association between delivery mode and SAMM was estimated from multivariate Poisson regression models. Of 5,055 women with planned vaginal delivery, 4,007 (79.3%) delivered both twins vaginally, 134 (2.6%) had cesarean for the second twin and 914 (18.1%) cesarean for both twins. Compared to vaginal delivery of both twins, the risk of SAMM was significantly higher after cesarean for the second twin (9.0% versus 4.5%; aRR 2.22, 95% CI 1.27–3.88) and for both twins (9.4% versus 4.5%, aRR 1.56, 95% CI 1.16–2.10). In twin pregnancies with planned vaginal delivery, cesarean deliveries for the second twin and for both twins are associated with higher risks of SAMM than vaginal delivery.

## Introduction

Twin pregnancies are increasingly frequent in developed countries and concern about 3% of all births in the United States and France^1,2^. Results of informative studies since 2013 have shown the absence of neonatal or maternal benefits associated with planned cesarean and thus encourage professionals and women to plan vaginal delivery^3–5^. Nonetheless, data are sparse about severe acute maternal morbidity according to the actual mode of delivery after vaginal delivery is planned for twin pregnancies. Furthermore, extrapolation of the results from singletons to twin pregnancies is difficult. Indeed, maternal morbidity may be increased because of the uterine overdistension in twin pregnancy and because unlike singleton pregnancies, planned vaginal delivery has three potential outcomes: vaginal or cesarean delivery of both twins, or cesarean for the second twin. Cesareans for the second twin, performed at full dilation and after the first twin has passed through the birth canal, possibly after intrauterine manoeuvres, are consequently at potentially high risk of maternal morbidity, including infection and postpartum hemorrhage. The reported rates of cesareans for the second twin range from 0.5% to more than 10%^5–10^ and for both twins from 19.6% to 43.8%^3,4^. These large rate ranges may reflect wide variations in practices related to heterogeneous indications for cesareans and suggest that some of these procedures are performed when vaginal delivery might have been possible. A better knowledge of the maternal risks associated with each of these three modes of delivery would usefully inform decisions during the management of labor in women with twin pregnancies.

Only a few studies have reported the risk of maternal complications, always as secondary outcomes, according to the actual mode of delivery in twin pregnancies with planned vaginal delivery. They are limited by their retrospective designs, long-past study periods and non-exhaustive definitions of acute maternal morbidity^9,11–13^. The risk of severe acute maternal morbidity associated with the mode of delivery for twin pregnancy after planned vaginal delivery thus remains unclear.

In this planned secondary analysis of the JUmeaux MODe d’Accouchement (JUMODA) cohort^3^, our aim was to assess in twin pregnancies with planned vaginal deliveries the association between the three possible modes of delivery and severe acute maternal morbidity.

## Materials and Methods

The national, observational, prospective, population-based cohort study of the mode of delivery of twin pregnancies (JUMODA: JUmeaux MODe d’Accouchement) conducted by Schmitz *et al*.^4^, took place in France from 10 February, 2014, through 1 March, 2015. All French maternity units performing more than 1,500 annual deliveries were invited to participate, and 176 of the 191 eligible units (92%) agreed. In women with vaginal delivery of the first twin, French guidelines recommend active management of second twin delivery, including early pushing or obstetrical manoeuvres, according to the fetal presentation and station, and the operator’s experience^14^.

Detailed information about the participating women and maternity units has been reported by Schmitz *et al*.^4^. Briefly, this cohort was specially designed to assess the effect of the mode of delivery on neonatal and maternal outcomes in twin pregnancies at or after 22 weeks of gestation (N = 8,823 women included). Immediately after delivery, obstetricians completed a detailed web-based questionnaire about the mode of delivery, indications for cesarean and details of delivery management. Research nurses collected data about maternal characteristics, medical history, pregnancy complications, maternal complications and neonatal health.

**Figure 1:**
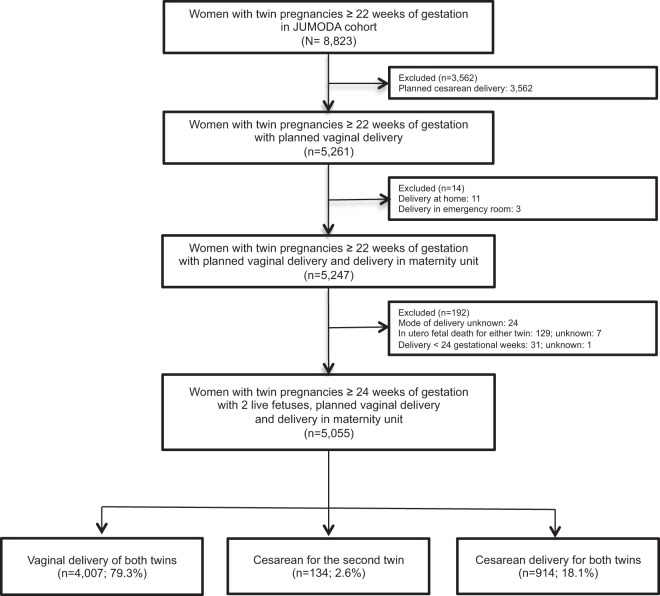
Flow chart.

For this planned secondary analysis, we excluded women with planned cesarean deliveries (n = 3,562), and deliveries at home or in the emergency room (n = 14). We also excluded women for whom the mode of delivery was unknown (n = 24) and those with in utero fetal death or medical termination of at least one of the two twins (n = 136) and delivery before 24 weeks of gestation (n = 32) (Fig. 1). Therefore, 5,055 women with planned vaginal deliveries were analysed (Fig. 1).

The primary outcome was a composite of severe acute maternal morbidity. This multicriteria definition was developed in a formal national Delphi expert consensus process for another study specifically conducted to study severe acute maternal morbidity and was already reported^15^. To include conditions involving severe health impairments, it combined diagnoses, organ dysfunctions and interventions, as recommended by WHO^16^. We used the same definition as in a previous analysis of the JUMODA cohort data^5^. Severe acute maternal morbidity was therefore defined as any one or more of the following: maternal death; severe postpartum hemorrhage defined by need for second line therapy, transfusion ≥4 units of packed red blood cells, uterine artery embolisation, vascular ligation, compressive uterine suture, emergency peripartum hysterectomy; pulmonary embolism; stroke or cerebral transient ischaemic attack; severe psychiatric disorder; cardiovascular or respiratory dysfunction, renal dysfunction (creatinine >1.47 mg/dl or oliguria <500 ml/24 h), neurological dysfunction (coma of any stage and duration), or haematological dysfunction (thrombocytopenia <50 000/mm^3^ in the absence of a chronic disorder, or acute anaemia <7 g/dl); emergency surgery in addition to the childbirth procedure, e.g., secondary hysterectomy, laparotomy for post-delivery complication; or admission to an intensive care unit. We purposely did not include third- and fourth-degree perineal lacerations or cervical lacerations in the composite maternal outcome unless they were associated with another criterion for severe acute maternal morbidity, as previously discussed^5^. This primary outcome was treated as a binary variable. We also analysed maternal infectious morbidity, defined as one or more of the following: endometritis, temperature ≥38.5 °C on two or more occasions within 24 hours, and positive haemoculture.

The exposure of interest was the mode of delivery in 3 categories: vaginal delivery of both twins (reference group), cesarean for the second twin after vaginal birth of the first, and cesarean for both twins during labor. Potential confounders determined from the literature included: maternal age, body mass index, parity and history of previous cesareans; characteristics of the current pregnancy, including *in vitro* fertilisation, pregnancy complications (defined as a binary variable by the presence of at least one of the following: hypertension, preeclampsia, insulin-treated diabetes, hospitalization for bleeding during 2^nd^ or 3^rd^ trimester, and twin-twin transfusion syndrome); characteristics of labor and delivery, including gestational age at delivery, spontaneous labor, oxytocin during labor, second-twin presentation; macrosomia (defined by a total birth weight of both twins> 90^th^ percentile of the distribution of birth weights in this cohort, i.e. 5800 g); and the hospital’s annual volume of twin deliveries.

We compared the characteristics of the women, pregnancies, labors, neonates, and hospitals according to the mode of delivery, based on Chi^2^ or Fisher exact tests for categorical variables and Student’s or Wilcoxon rank sum tests for quantitative variables, as appropriate. To assess the association between the mode of delivery and severe acute maternal morbidity, while controlling for confounding by indication, we used multivariate Poisson regression modelling to adjust for prognostic covariates with a random intercept model to take variability between centres into account^16^. We also compared the maternal infectious morbidity rate between the three groups. The differences in severe acute maternal morbidity rates by mode of delivery were tested according to the underlying causal condition of this morbidity by differentiating on the one hand severe postpartum hemorrhage from on other side other underlying causal conditions.

The proportion of women with missing data for any covariate ranged from 0% to 4.0%; the 4,744 (94.0%) women with full data had characteristics similar to those of the women with missing data. We used multiple imputation-chained equations to impute missing data and generated 6 independent imputation data sets.

In a sensitivity analysis, we assessed the association between the mode of delivery and severe acute maternal morbidity after application of the selection criteria of the Twin Birth Study^3^ (TBS-like population – Table S2) and therefore excluded women with a gestational age less than 32 weeks 0 days, a first twin in non-cephalic presentation, an estimated fetal weight of either twin less than 1,500 grams or more than 4,000 grams, monoamniotic twins, fetal reduction at or after 13 weeks of gestation, fetal anomalies, or a second twin substantially larger than the first twin^2^. This TBS-like population thus comprised 3,977 women (Table S2).

All tests were two-sided with *P* values <0.05 defined as statistically significant. STATA 13 software (StataCorp LP, College Station, Texas, USA) was used for the descriptive and multivariate analyses.

The procedures of the study received ethical approval from the National Data Protection Authority (DR-2013-528), the consultative committee on the treatment of information on personal health data for research purposes (13–298), and the committee for the protection of people participating in biomedical research of Paris Ile-de-France 7 (PP-13-014). They approved that this observational study waived the need to obtain informed consent according to the French law.

## Results

Our study population included 5,055 women with planned vaginal delivery: 4,007 (79.3%) gave birth to both twins vaginally, 134 (2.6%) had a cesarean for the second twin and 914 (18.1%) a cesarean for both twins (Fig. 1).

Compared to women with vaginal delivery of both twins, those with cesareans for the second twin had similar maternal and pregnancy characteristics but lower rates of labor induction and of oxytocin use during labor, as well as higher rates of delivery before 37 weeks of gestation (Tables 1 and S1). The main indication for cesarean for the second twin was the failure of intrauterine manoeuvres for this twin’s vaginal delivery (35.8%) (Table 2).

**Table 1:** Maternal, pregnancy, labor and delivery characteristics of the main population according to mode of delivery.

	Vaginal delivery of both twins	Cesarean delivery for the second twin	*P*	Cesarean delivery for both twins	*P*
	n = 4,007	n = 134		n = 914	
	n (%)	n (%)		n (%)	
Age (mean ± SD, years)	31.1 ± 5	31.2 ± 5	0.832	32.2 ± 6	<0.001
<30	1,499 (37.4)	50 (37.3)	0.992	310 (34.0)	<0.001
[30–34]	1,540 (38.4)	51 (38.1)		313 (34.2)	
≥35	968 (24.2)	33 (24.6)		291 (31.8)	
BMI before pregnancy (mean ± SD, Kg.m-2)	23.7 ± 4.7	24.3 ± 5.4	0.193	23.7 ± 4.6	0.905
<18.5	257 (6.7)	9 (7.3)	0.470	60 (6.8)	0.932
[18.5–25[	2,432 (63.4)	71 (57.3)		573 (64.4)	
[25–30[	763 (19.9)	27 (21.8)		169 (18.9)	
≥30	387 (10.1)	17 (13.7)		88 (9.9)	
Parity and previous cesareans			0.745		<0.001
Nulliparous	1,704 (42.6)	55 (41.0)		644 (70.5)	
Parous with no previous cesarean	2,158 (54.0)	73 (54.5)		208 (22.8)	
Parous with previous cesarean	133 (3.3)	6 (4.5)		61 (6.7)	
*In vitro* fertilisation	776 (19.5)	31 (23.1)	0.294	297 (32.6)	<0.001
Pregnancy complications	883 (22.1)	28 (21.1)	0.773	282 (30.9)	<0.001
Gestational age at delivery (weeks days) (mean)	36 1/7	35 4/7	0.024	36 5/7	<0.001
<32 0/7	266 (6.6)	14 (10.4)	0.124	39 (4.3)	<0.001
32 0/7-36 6/7	1,783 (44.5)	66 (49.3)		334 (36.6)	
≥37 0/7	1,955 (48.8)	54 (40.3)		540 (59.1)	
Induction of labor	1,687 (42.1)	38 (28.4)	0.002	571 (62.6)	<0.001
Oxytocin during labor	2,778 (70.2)	82 (61.7)	0.035	672 (75.4)	0.002
Non-cephalic second-twin presentation	1,607 (40.1)	64 (47.8)	0.077	378 (41.5)	0.451
Macrosomia	447 (11.2)	14 (10.6)	0.837	138 (15.1)	0.001
Annual number of twin deliveries			0.066		0.038
<50	1,360 (33.9)	57 (42.5)		272 (29.8)	
[50–99]	1,098 (27.4)	37 (27.6)		255 (27.9)	
≥100	1,549 (38.7)	40 (29.9)		387 (42.3)	

BMI: Body mass index.

SD: standard deviation.

**Table 2:** Indications for cesarean deliveries.

	Indications n (%)
**Cesarean for second twin (n = 134) (not exclusive)**	
Failure of intrauterine manoeuvres	48 (35.8)
No descent of the fetal presentation	43 (32.1)
Bradycardia	41 (30.6)
Cervical retraction	40 (29.9)
Prolapse of an arm	16 (11.9)
Prolapse of umbilical cord	12 (9.0)
Non-cephalic presentation	8 (6.0)
Uterine hypertonia	5 (3.7)
Placental abruption	3 (2.2)
Other	18 (13.4)
**Cesarean for both twins (n = 914) (not exclusive)**	
Non-reassuring fetal heart rate monitoring	322 (35.3)
Labor arrest	320 (35.0)
Failure of labor induction	131 (14.4)
Non-engagement	80 (8.8)
Labor arrest and non-reassuring fetal heart rate monitoring	38 (4.2)
Bleeding	7 (0.8)
Other	74 (8.0)

Women with a cesarean for both twins were older and more often nulliparous than those with a vaginal delivery of both twins; they also had higher rates of previous cesarean deliveries, *in vitro* fertilisation and pregnancy complications, as well as more frequent labor induction and oxytocin during labor (Tables 1 and S1). They also gave birth at a later gestational age to larger neonates. The main indications for cesarean delivery for both twins were a non-reassuring fetal heart rate (35.3%) and labor dystocia (35.0%) (Table 2).

Compared to the vaginal group, the risk of severe acute maternal morbidity was higher among women with cesareans for the second twin (12/134 – 9.0% – compared to 179/4,007 – 4.5%; aRR: 2.22, 95% CI 1.27-3.88), and among women with cesareans for both twins (86/914 – 9.4% – compared to 179/4,007 – 4.5%; aRR: 1.56, 95% CI 1.16-2.10) (Table 3). These rates did not differ among women with cesareans for second twins and for both twins (12/134 – 9.0% – compared to 86/914 – 9.4%; *P* = 0.866).

**Table 3:** Association between mode of delivery and severe acute maternal morbidity in the overall population, and underlying causal conditions of severe acute maternal morbidity in each group.

	Vaginal delivery of both twins	Cesarean delivery for the second twin	Crude RR (95% CI)	Adjusted RR* (95% CI)	Cesarean delivery for both twins	Crude RR (95% CI)	Adjusted RR* (95% CI)
	n = 4,007 n (%)	n = 134 n (%)			n = 914 n (%)		
Severe acute maternal morbidity	179 (4.5)	12 (9.0)	2.00 (1.15–3.56)	2.22 (1.27–3.98)	86 (9.4)	2.11 (1.65–2.70)	1.56 (1.16–2.10)
Death	0 (0.0)	0 (0.0)			0 (0.0)		
Severe postpartum hemorrhage	154 (3.9)	11 (8.2)^†^			68 (7.5)^**^		
Blood transfusion ≥4 units of red blood cells	31 (0.8)	2 (1.5)			16 (1.8)		
Uterine artery embolisation	24 (0.6)	2 (1.5)			4 (0.4)		
Vascular ligations, compressive uterine sutures	9 (0.2)	2 (1.5)			25 (2.7)		
Hysterectomy	4 (0.1)	1 (0.7)			4 (0.4)		
Other underlying causal conditions of SAMM	57 (1.4)	4 (3.0)^††^			39 (4.3)^**^		
Pulmonary embolism	2 (0.1)	0 (0.0)			3 (0.3)		
Stroke or cerebral transient ischaemic attack	0 (0.0)	0 (0.0)			0 (0.0)		
Severe psychiatric disorders	1 (0.02)	0 (0.0)			0 (0.0)		
Cardiovascular dysfunction	2 (0.1)	0 (0.0)			1 (0.1)		
Respiratory dysfunction	1 (0.03)	0 (0.0)			1 (0.1)		
Renal dysfunction	8 (0.2)	1 (0.7)			8 (0.9)		
Haematological dysfunction	25 (0.6)	1 (0.8)			13 (1.4)		
Neurological dysfunction	0 (0.0)	0 (0.0)			0 (0.0)		
Emergency surgery	2 (0.1)	3 (2.2)			14 (1.5)		
Admission to an intensive care unit	30 (0.8)	2 (1.5)			16 (1.8)		
Infectious morbidity^ǂ^	27 (0.7)	3 (2.2)^ǂǂ^			13 (1.4)^ǂǂǂ^		
Endometritis	7 (0.2)	3 (2.2)			5 (0.5)		
Temperature ≥38.5 °C on ≥2 occasions in 24 h	14 (0.4)	1 (0.8)			9 (1.0)		
Positive hemoculture	12 (0.3)	1 (0.8)			4 (0.4)		

*Adjusted for maternal age, body mass index, parity, and previous cesarean delivery, *in vitro* fertilization, pregnancy complication, gestational age at delivery, induction of labor, oxytocin during labor, second twin presentation, macrosomia, annual number of twin deliveries per center.

For all comparisons, the reference group is vaginal delivery of both twins.

^**^P = < 0.001.

^†^*P* = 0.011.

^††^*P* = 0.140.

^ǂǂ^*P* = 0.036.

^ǂǂǂ^*P* = 0.023.

^ǂ^These outcomes were not components of the composite primary outcome.

Severe postpartum hemorrhage was the most frequent contributor to severe acute maternal morbidity in all three groups. When severe acute maternal morbidity was analysed by underlying causal condition and compared to the vaginal group, cesareans for second twins and for both twins were associated with higher rates of both severe postpartum hemorrhage and severe acute maternal morbidity from all other underlying causal conditions combined (Table 3). Similarly, compared with the vaginal group, infectious morbidity (not limited to severe cases) was more frequent both in women with cesareans for the second twin (2.2% compared to 0.7%, *P* = 0.036) and with cesareans for both twins (1.4% compared to 0.7%, *P* = 0.023).

In the Twin Birth Study-like population, 3,199 (80.4%) women had vaginal deliveries of both twins, 102 (2.6%) had cesareans for the second twins and 676 (17.0%) cesareans for both twins (Fig. S1). The characteristics of women in these three groups were similar to those of the overall population (Table S2). Compared with the vaginal group, the risk of severe acute maternal morbidity was higher both in women with cesareans for second twins (9/102 – 8.8% – versus 150/3199 – 4.7%; aRR 2.06, 95% CI 1.07-3.98); and for both twins (66/676 – 9.8% –versus 150/3199 – 4.7%; aRR 1.55, 95% CI 1.11–2.17) (Table S3).

## Discussion

In this prospective population-based study of women with twin pregnancies and planned vaginal deliveries, cesareans for second twins and for both twins were associated with higher risks of severe acute maternal morbidity than vaginal delivery of both twins. Severe postpartum hemorrhage was the main contributor to this morbidity.

Strengths of our study are the following. It was population-based and included a large number of cesareans for the second twin. The planning of this analysis during the design of the JUMODA study allowed the prospective collection of the data needed to precisely characterize the risk of severe acute maternal morbidity, while adjusting for numerous potential confounders. Finally, the results of the primary analysis were validated by the sensitivity analysis in the Twin Birth Study-like population, a population of women for whom planned vaginal delivery is consensually accepted.

This study was limited by the fact that we could not report the specific rate of severe maternal sepsis, although sepsis cases were included in our definition of severe acute maternal morbidity through the organ dysfunction or admission to intensive care criteria. However, using a broad definition of maternal infection as in previous studies, we similarly found an increased rate of this morbidity in cesareans for both twins and for second twins. Moreover, the rarity of the causes of severe acute maternal morbidity other than severe postpartum hemorrhage limited specific analyses of their associations with mode of delivery. Finally, because the JUMODA cohort comes only from maternity hospitals with more than 1500 annual deliveries, the generalisability of our results to the hospitals performing fewer deliveries may be limited.

A previous analysis conducted from the same twin cohort showed that there was no association between the planned mode of delivery, vaginal or caesarean, and severe acute maternal morbidity, except for women aged 35 years or more who were at greater risk of such morbidity after planned cesarean delivery^5^. Although we did not conduct a formal comparison with planned cesareans, we found, as expected, that vaginal delivery for both twins was the mode of delivery associated with the lowest risk of severe acute maternal morbidity.

Our results provide new and important information on the association between the actual mode of delivery in twin pregnancies and severe acute maternal morbidity, in view of the scarcity of available data on this topic^10,12,13^. Moreover, as the study of maternal morbidity was not the primary objective in these previous analyses, they were not designed to address this question. Therefore, they lacked strategies to take confounding factors into account and had questionable control groups^10,12,13^. The only previous study comparing all three modes of delivery for women with a planned vaginal delivery of twins^13^ also found a higher risk of maternal morbidity associated with cesareans for second twins and for both twins than with vaginal delivery of both twins. These results were limited by their retrospective design, data from the 1980s and 1990s, a definition of maternal morbidity that included few if any severe events, a limited number of cesareans for the second twin, and a lack of adjustment for confounders. The multicentre retrospective study of Wenckus *et al*. comparing maternal and neonatal outcomes in twins undergoing a trial of labor versus prelabor cesarean did not differentiate the 57 cesareans for second twins from the cesareans during labor for both twins and compared the actual mode of delivery to planned cesarean^10^. The other study that analysed maternal morbidity according to mode of delivery for women for whom vaginal delivery was planned limited its comparison to cesareans for both twins and cesareans for second twins, thereby omitting the principal clinical alternative: vaginal delivery for both twins^12^. They found a higher risk of endometritis for cesareans for the second twin, as we did.

Our study found that severe postpartum hemorrhage was the principal driver of the higher risk of severe acute maternal morbidity associated with cesareans during labor for twin pregnancies, although some previous studies have reported that infection is the main contributor to maternal morbidity^9,10^. The definitions of infection and postpartum hemorrhage used in previous studies, which did not focus on severe cases, may explain this difference. The increased risk of severe postpartum hemorrhage observed in our study is consistent with results in singleton pregnancies^17–19^. The higher risk in singletons is especially marked for cesareans during labor and for cesareans during the second stage of labor^19,20^– both conditions met by cesareans for second twins.

The results of this study can have implications for practitioners and may be useful in guiding the management of twin delivery by providing additional information about the maternal risks related to the actual mode of delivery after planned vaginal delivery. They show that cesarean delivery for the second twin, which is feared because of a potential excess risk of complications, is not in fact at higher maternal risk than cesarean delivery for both twins during labor.

These results highlight the importance of achieving vaginal delivery for both twins, to limit the occurrence of severe maternal morbidity events. The analysis of indications for cesareans for both twins — half of which are performed for non-reassuring fetal heart rate or for labor dystocia — offers insights for identifying cesareans that could be avoided. Possibly, particular attention to accurate fetal heart rate analysis and management of non-optimal cervical dilation could limit the number of cesareans. Likewise, as our results show that a cesarean for the second twin is performed in half of the cases for failure of manoeuvres, improved training in the active management of the second twin could increase the rate of successful vaginal deliveries for both twins.

## Conclusion

A large majority of women with twin pregnancies and planned vaginal deliveries gave birth to both twins vaginally, which is the situation associated with the lowest risk of severe acute maternal morbidity. Our results could help obstetricians to inform women with twin pregnancies and to take decisions about management during their labor, and provide support for auditing indications for cesarean deliveries in twins.

## Supplementary information

SUPPLEMENTARY INFORMATION.

## Data Availability

The dataset analysed is not publicly available.
