# Supplementary material for: Severe maternal morbidity by mode of delivery in women with twin pregnancy and planned vaginal delivery
Source: Sci Rep. 2020 Mar 18;10:4944. doi: 10.1038/s41598-020-61720-w (PMC7080743; doi:10.1038/s41598-020-61720-w)
Supplement: Supplementary file 1 — SUPPLEMENTARY INFORMATION. [file 41598_2020_61720_MOESM1_ESM.pdf]

# Severe maternal morbidity by mode of delivery in women with twin pregnancy and planned vaginal delivery

Authors: Diane Korb, Catherine Deneux-Tharaux, François Goffinet, Thomas Schmitz

## SUPPLEMENTARY INFORMATIONS

Figure S1: Flow chart for the Twin Birth Study-like population

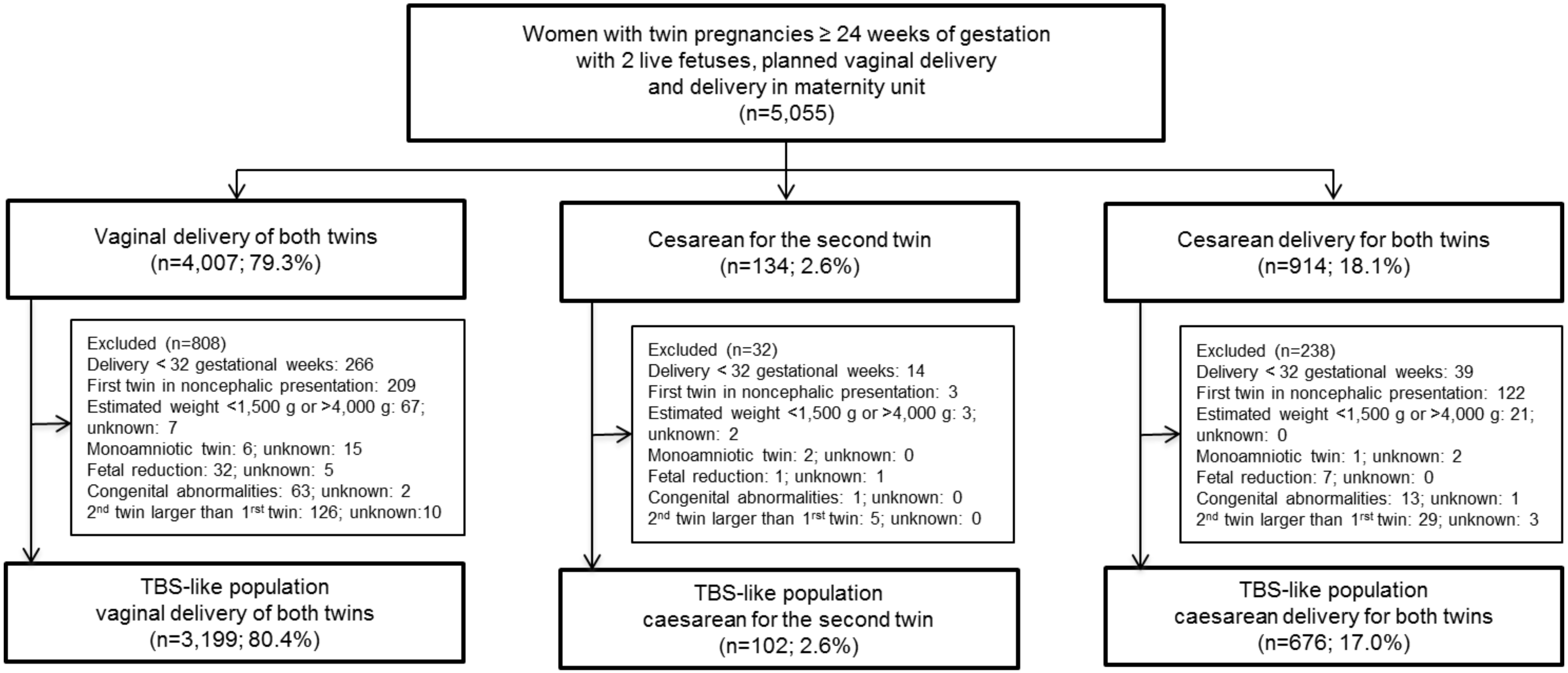


TBS, Twin Birth Study

Table S1: Details of maternal, pregnancy, labor and delivery characteristics of the main population according to mode of delivery

| | Vaginal delivery of both twins | Cesarean delivery for the second twin | *P* | Cesarean delivery for both twins | *P* |
|---|---|---|---|---|---|
| | n=4,007 | n=134 | | n=914 | |
| | n (%) | n (%) | | n (%) | |
| Age (mean ± SD, years) | 31.1±5 | 31.2±5 | 0.832 | 32.2±6 | <0.001 |
| <30 | 1,499 (37.4) | 50 (37.3) | 0.992 | 310 (34.0) | <0.001 |
| [30-34] | 1,540 (38.4) | 51 (38.1) | | 313 (34.2) | |
| ≥35 | 968 (24.2) | 33 (24.6) | | 291 (31.8) | |
| BMI before pregnancy (mean±SD, Kg.m-2) | 23.7±4.7 | 24.3±5.4 | 0.193 | 23.7±4.6 | 0.905 |
| <18.5 | 257 (6.7) | 9 (7.3) | 0.470 | 60 (6.8) | 0.932 |
| [18.5-25[ | 2,432 (63.4) | 71 (57.3) | | 573 (64.4) | |
| [25-30[ | 763 (19.9) | 27 (21.8) | | 169 (18.9) | |
| ≥30 | 387 (10.1) | 17 (13.7) | | 88 (9.9) | |
| Parity and previous cesareans | | | 0.745 | | <0.001 |
| Nulliparous | 1,704 (42.6) | 55 (41.0) | | 644 (70.5) | |
| Parous with no previous cesarean | 2,158 (54.0) | 73 (54.5) | | 208 (22.8) | |
| Parous with previous cesarean | 133 (3.3) | 6 (4.5) | | 61 (6.7) | |
| Geographic region of birth | | | 0.757 | | 0.423 |
| Europe | 2,958 (82.4) | 104 (86.0) | | 642 (80.4) | |
| North Africa | 377 (10.5) | 11 (9.1) | | 85 (10.7) | |
| Africa other | 201 (5.6) | 5 (4.1) | | 53 (6.6) | |
| Other | 55 (1.5) | 1 (0.8) | | 18 (2.3) | |
| Smoker | 563 (14.6) | 25 (20.0) | 0.091 | 118 (13.3) | 0.343 |
| History of diabetes | 18 (0.5) | 0 (0.0) | 0.436 | 7 (0.8) | 0.225 |
| History of hypertension | 27 (0.7) | 1 (0.7) | 0.922 | 10 (1.1) | 0.184 |
| In vitro fertilisation | 776 (19.5) | 31 (23.1) | 0.294 | 296 (32.5) | <0.001 |
| Fetal reduction ≥ 13 weeks gestation | 37 (0.9) | 1 (0.8) | 0.836 | 11 (1.2) | 0.442 |
| Chorionicity | | | 0.164 | | 0.287 |
| Dichorionic | 3,200 (80.2) | 112 (84.8) | | 754 (82.7) | |
| Monochorionic/diamnionic | 783 (19.6) | 20 (15.2) | | 157 (17.2) | |
| Monochorionic/monoamnionic | 7 (0.2) | 0 (0.0) | | 1 (0.1) | |
| Pregnancy complications | 883 (22.1) | 28 (21.1) | 0.773 | 282 (30.9) | <0.001 |
| Hypertension | 155 (3.9) | 5 (3.8) | | 61 (6.7) | |

| | | | | | |
|---|---|---|---|---|---|
| Preeclampsia | 230 (5.8) | 6 (4.5) | | 123 (13.5) | |
| Bleeding | 34 (0.9) | 2 (1.5) | | 13 (1.4) | |
| Placental abruption | 2 (0.1) | 0 (0.0) | | 2 (0.2) | |
| IUGR for either twin | 413 (10.3) | 14 (10.5) | | 112 (12.3) | |
| Insulin-treated diabetes | 112 (2.8) | 4 (3.0) | | 33 (3.6) | |
| Placenta praevia | 8 (0.2) | 0 (0.0) | | 1 (0.1) | |
| Malformation for either twin | 81 (2.0) | 2 (1.5) | | 21 (2.3) | |
| Twin-to-twin transfusion syndrome | 56 (1.4) | 0 (0.0) | | 9 (1.0) | |
| Premature rupture of membranes | 390 (9.8) | 14 (10.6) | 0.750 | 71 (7.8) | 0.064 |
| Preterm labor | 1,550 (38.8) | 48 (36.4) | 0.577 | 255 (27.9) | <0.001 |
| Antenatal corticosteroids | 1,792 (44.9) | 53 (39.8) | 0.250 | 329 (36.1) | <0.001 |
| Induction of labor | 1,685 (42.1) | 38 (28.4) | 0.002 | 571 (62.7) | <0.001 |
| Oxytocin during labor | 2,776 (70.2) | 82 (61.7) | 0.034 | 670 (75.4) | 0.002 |
| Non-cephalic second-twin presentation | 1,604 (40.1) | 64 (47.8) | 0.075 | 376 (41.4) | 0.483 |
| Gestational age at delivery (weeks days) (mean) | 36 1/7 | 35 4/7 | 0.024 | 36 5/7 | <0.001 |
| <32 0/7 | 266 (6.6) | 14 (10.4) | 0.124 | 39 (4.3) | <0.001 |
| 32 0/7-36 6/7 | 1,783 (44.5) | 66 (49.3) | | 334 (36.6) | |
| ≥ 37 0/7 | 1,955 (48.8) | 54 (40.3) | | 540 (59.1) | |
| Intertwin delivery interval (median (Q1-Q3), minutes) | 6 [4-9] | 19 [9-58] | <0.001 | 1 [1-2] | <0.001 |
| General anaesthesia at 2nd twin delivery | 30 (0.8) | 37 (27.6) | <0.001 | 51 (5.6) | <0.001 |
| Instrumental vaginal delivery for 1st twin | 751 (18.8) | 32 (23.9) | 0.137 | NA | NA |
| Instrumental vaginal delivery for 2nd twin | 602 (15.0) | 0 (0.0) | | NA | NA |
| Episiotomy | 905 (22.7) | 28 (20.9) | 0.630 | NA | NA |
| Birth weight 1st twin (mean±SD, grams) | 2,381.7±524.0 | 2,276.6±559.7 | 0.023 | 2,488.1±510.6 | <0.001 |
| Birth weight 2nd twin (mean±SD, grams) | 2,336.4±516.3 | 2,255.1±568.1 | 0.076 | 2,413.5±504.5 | <0.001 |
| Macrosomia | 447 (11.2) | 14 (10.6) | 0.837 | 138 (15.1) | 0.001 |
| Annual number of twin deliveries | | | 0.066 | | 0.038 |
| <50 | 1,360 (33.9) | 57 (42.5) | | 272 (29.8) | |
| [50-99] | 1,098 (27.4) | 37 (27.6) | | 255 (27.9) | |
| ≥100 | 1,549 (38.7) | 40 (29.9) | | 387 (42.3) | |
| University hospital | 1,883 (47.0) | 52 (38.8) | 0.062 | 471 (51.5) | 0.013 |
| Level of care | | | 0.005 | | 0.043 |

| | | | | | |
|---|---|---|---|---|---|
| I | 73 (1.8) | 7 (5.2) | | 18 (2.0) | |
| II | 1,488 (37.2) | 57 (42.5) | | 299 (32.7) | |
| III | 2,446 (61.0) | 70 (52.2) | | 597 (65.3) | |

BMI: Body mass index

SD: standard deviation

IUGR: in utero growth restriction

Table S2: Maternal, pregnancy, labor and delivery characteristics of the Twin Birth Study-like population according to mode of delivery

| | **Vaginal delivery of both twins** | **Cesarean delivery for the second twin** | ***P*** | **Cesarean delivery for both twins** | ***P*** |
|---|---|---|---|---|---|
| | **n=3,199** | **n=102** | | **n=676** | |
| | **n (%)** | **n (%)** | | **n (%)** | |
| Age (mean ± SD, years) | 31.2±5.0 | 31.1±4.6 | 0.911 | 32.3±5.7 | <0.001 |
| <30 | 1,165 (36.4) | 36 (35.3) | 0.482 | 231 (34.3) | 0.001 |
| [30-34] | 1,241 (38.8) | 45 (44.1) | | 229 (33.8) | |
| ≥35 | 792 (24.8) | 21 (20.6) | | 216 (31.9) | |
| BMI before pregnancy (mean±SD, $Kg.m^2$) | 23.6±4.7 | 23.9±5.3 | 0.518 | 23.7±4.5 | 0.463 |
| <18.5 | 209 (6.8) | 7 (7.4) | 0.892 | 47 (7.1) | 0.687 |
| [18.5-25[ | 1,986 (64.4) | 58 (61.1) | | 411 (62.3) | |
| [25-30] | 596 (19.3) | 19 (20.0) | | 140 (21.1) | |
| ≥30 | 295 (9.5) | 11 (11.6) | | 62 (9.4) | |
| Geographic region of birth | | | 0.443 | | 0.829 |
| France | 2,254 (78.6) | 81 (86.2) | | 457 (77.7) | |
| Europe | 110 (3.8) | 2 (2.1) | | 22 (3.7) | |
| North Africa | 305 (10.6) | 8 (8.5) | | 60 (10.2) | |
| Africa other | 156 (5.4) | 2 (2.1) | | 38 (6.5) | |
| Other | 43 (1.5) | 1 (1.1) | | 11 (1.9) | |
| Parity and previous cesareans | | | 0.822 | | <0.001 |
| Nulliparous | 1,327 (41.6) | 38 (37.3) | | 487 (72.2) | |
| Parous with no previous cesarean | 1,769 (55.4) | 60 (58.8) | | 144 (21.3) | |
| Parous with previous cesarean | 98 (3.1) | 4 (3.9) | | 44 (6.5) | |
| Smoker | 439 (14.2) | 18 (19.1) | 0.178 | 84 (12.8) | 0.326 |

| | | | | | |
|---|---|---|---|---|---|
| History of diabetes | 15 (0.5) | 0 (0.0) | 0.488 | 4 (0.6) | 0.674 |
| History of hypertension | 20 (0.6) | 1 (1.0) | 0.657 | 6 (0.9) | 0.444 |
| In vitro fertilisation | 615 (19.3) | 21 (20.6) | 0.746 | 228 (33.8) | <0.001 |
| Pregnancy complications | 605 (18.9) | 20 (19.6) | 0.865 | 178 (26.4) | <0.001 |
| Hypertension | 128 (4.0) | 4 (3.9) | | 48 (7.1) | |
| Preeclampsia | 187 (5.8) | 6 (5.9) | | 98 (14.5) | |
| Placental abruptio | 2 (0.1) | 0 (0.0) | | 1 (0.2) | |
| IUGR for either twin | 286 (8.9) | 11 (10.8) | | 66 (9.8) | |
| Insulin-treated diabetes | 84 (2.6) | 2 (2.0) | | 20 (3.0) | |
| Placenta previa | 6 (0.2) | 0 (0.0) | | 1 (0.2) | |
| Twin-to-twin transfusion syndrome | 36 (1.1) | 0 (0.0) | | 3 (0.4) | |
| Premature rupture of membranes | 241 (7.5) | 9 (8.8) | 0.629 | 44 (6.5) | 0.354 |
| Preterm labor | 1,141 (35.7) | 34 (33.3) | 0.623 | 162 (24.0) | <0.001 |
| Antenatal corticosteroids | 1,293 (40.5) | 37 (36.3) | 0.396 | 206 (30.6) | <0.001 |
| Induction of labor | 1,489 (46.6) | 35 (34.3) | 0.015 | 464 (68.9) | <0.001 |
| Oxytocin during labor | 2,297 (72.6) | 67 (65.7) | 0.122 | 519 (78.8) | 0.001 |
| Noncephalic second twin presentation | 1,209 (37.8) | 46 (45.1) | 0.136 | 271 (40.2) | 0.246 |
| Gestational age at delivery (gestational weeks) (mean) | 36 6/7 | 36 4/7 | 0.169 | 37 2/7 | <0.001 |
| 32 0/7-36 6/7 | 1,454 (45.5) | 55 (53.9) | 0.302 | 221 (32.6) | <0.001 |
| ≥ 37 0/7 | 1,745 (54.5) | 47 (46.1) | | 455 (67.4) | |
| Intertwin delivery interval, Intertwin delivery interval (med (Q1-Q3), minutes) | 6 [4-10] | 18 [13-25] | <0.001 | 1 [1-2] | <0.001 |
| General anaesthesia at 2nd twin delivery | 16 (0.5) | 27 (26.5) | <0.001 | 32 (4.7) | <0.001 |
| Instrumental vaginal delivery for 1rst twin | 650 (20.3) | 31 (30.4) | 0.013 | NA | NA |
| Instrumental vaginal delivery for 2nd twin | 529 (16.5) | 0 (0.0) | | NA | NA |
| Episiotomy | 759 (23.8) | 25 (24.5) | 0.863 | NA | NA |

| | | | | | |
|---|---|---|---|---|---|
| Birth weight 1st twin (mean±SD, grams) | 2,503.0±408.6 | 2,456.1±394.7 | 0.253 | 2,605.2±406.2 | <0.001 |
| Birth weight 2nd twin (mean±SD, grams) | 2,434.1±409.3 | 2,382.6±401.0 | .211 | 2,500.9±418.3 | <0.001 |
| Annual number of twin deliveries | | | .096 | | .003 |
| <50 | 1,185 (37.0) | 48 (47.1) | | 214 (31.7) | |
| [50-99] | 869 (27.2) | 26 (25.5) | | 174 (25.7) | |
| ≥100 | 1,145 (35.8) | 28 (27.5) | | 288 (42.6) | |
| University hospital | 1,374 (43.0) | 37 (36.3) | 0.180 | 325 (48.1) | 0.015 |
| Level of care | | | 0.078 | | 0.019 |
| I | 57 (1.8) | 4 (3.9) | | 14 (2.1) | |
| II | 1,312 (41.0) | 49 (48.0) | | 238 (35.2) | |
| III | 1,830 (57.2) | 49 (48.0) | | 424 (62.7) | |

BMI: Body mass index
SD: standard deviation
IUGR: in utero growth restriction

Table S3: Association between mode of delivery and severe acute maternal morbidity in the Twin Birth Study-like population and underlying causal conditions of severe acute maternal morbidity in each group

| | **Vaginal delivery of both twins** | **Cesarean delivery for the second twin** | **Crude RR (95% CI)** | **Adjusted RR* (95% CI)** | **Cesarean delivery for both twins** | **Crude RR (95% CI)** | **Adjusted RR* (95% CI)** |
|---|---|---|---|---|---|---|---|
| | **n=3,199** | **n=102** | | | **n=676** | | |
| | **n (%)** | **n (%)** | | | **n (%)** | | |
| Severe maternal morbidity | 150 (4.7) | 9 (8.8) | 1. 9 (1.0-3.6) | 2.1 (1.1-4.0) | 66 (9.8) | 2.1 (1.6-2. 8) | 1.6 (1.1-2.2) |
| Death | 0 (0.0) | 0 (0.0) | | | 0 (0.0) | | |
| Severe postpartum haemorrhage | 129 (4.0) | 9 (8.8) | | | 53 (7.9) | | |
| Blood transfusion ≥4 RBC | 25 (0.8) | 2 (2.0) | | | 14 (2.1) | | |
| Uterine artery embolisation | 19 (0.6) | 2 (2.0) | | | 4 (0.6) | | |
| Vascular ligation, compressive uterine sutures | 8 (0.3) | 1 (1.0) | | | 22 (3.3) | | |
| Hysterectomy | 4 (0.1) | 1 (1.0) | | | 4 (0.6) | | |
| Pulmonary embolism | 1 (0.03) | 0 (0.0) | | | 2 (0.3) | | |
| Stroke or cerebral transient ischaemic attack | 0 (0.0) | 0 (0.0) | | | 0 (0.0) | | |
| Severe psychiatric disorders | 1 (0.03) | 0 (0.0) | | | 0 (0.0) | | |
| Cardiovascular dysfunction | 2 (0.1) | 0 (0.0) | | | 1 (0.2) | | |
| Respiratory dysfunction | 1 (0.03) | 0 (0.0) | | | 1 (0.2) | | |
| Renal dysfunction | 7 (0.2) | 1 (1.0) | | | 7 (1.0) | | |
| Haematological dysfunction | 23 (0.7) | 1 (1.0) | | | 9 (1.4) | | |
| Neurological dysfunction | 0 (0.0) | 0 (0.0) | | | 0 (0.0) | | |
| Emergency surgery | 2 (0.1) | 3 (2.9) | | | 12 (1.8) | | |
| Admission to an intensive care unit | 22 (0.7) | 1 (1.0) | | | 15 (2.2) | | |

RBC: units of packed red blood cells
*Adjusted for maternal age, body mass index, parity, and previous cesarean delivery, in vitro fertilization, pregnancy complication, gestational age at delivery, induction of labor, oxytocin during labor, second twin presentation, macrosomia, annual number of twin deliveries per center
